# Glial Control of Synapse Number in Healthy and Diseased Brain

**DOI:** 10.3389/fncel.2019.00042

**Published:** 2019-02-13

**Authors:** Eunbeol Lee, Won-Suk Chung

**Affiliations:** Department of Biological Sciences, Korea Advanced Institute of Science and Technology (KAIST), Daejeon, South Korea

**Keywords:** astrocytes, microglia, synapse loss, Alzheimer’s disease, neurodegenerative diseases

## Abstract

Glial cells are emerging as crucial players that mediate development and homeostasis of the central nervous system (CNS). In particular, glial cells are closely associated with synapses, and control synapse formation, function, plasticity, and elimination during the stages of development and adulthood. Importantly, it is now increasingly evident that abnormal glial function can be an active inducer of the initiation and progression of various neurodegenerative diseases. Here, we discuss recent developments on the physiological roles of glial cells in the brain, and propose that synapse loss, which is a common characteristic of several neurodegenerative diseases, can be initiated by mis-regulation of normal glial function.

## Introduction

Glial cells, non-neuronal central nervous system (CNS) cells, are not simply passive support cells for neurons, but active players in neuronal network formation and information processing. The fine processes of astrocytes and microglia closely associate with synapses and affect synapse formation and elimination during development. Even after initial establishment of a neural circuit, glial cells continue to participate in regulation of synapse number and modulation of synaptic function and plasticity. Due to their crucial roles in synapses, it is not surprising that mis-regulation of glial function has recently been revealed to be one of the initiating factors for onset and progression of various neurodegenerative diseases (Phatnani and Maniatis, 2015; Liddelow and Barres, 2017; Li and Barres, 2018).

Synapse loss is one of the most common, and earliest, pathophysiological features of neurodegenerative disease progression. In Alzheimer’s disease (AD), where amyloid β (Aβ) plaques accumulate in the brain, surrounded by reactive glial cells, synapses are weakened and undergo specific loss long before neuronal cell death (Perry and Holmes, 2014; Phatnani and Maniatis, 2015; Ransohoff, 2016). Previously, it was thought that this early synapse loss was mainly due to the direct effects of Aβ oligomers and fibrils on neuronal Aβ receptors or secondary neuroinflammation. However, recent studies have revealed that it is reactive glial cells that actually drive the initial synapse loss in AD (Hong et al., 2016; Shi Q. et al., 2017; Dejanovic et al., 2018; Litvinchuk et al., 2018).

**Figure 1:**
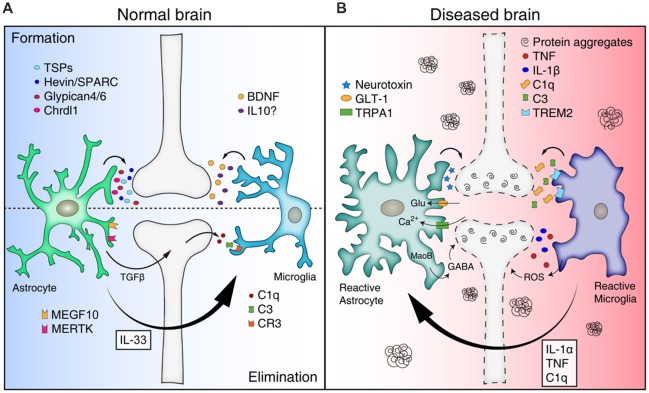
Mis-regulation of glial function can lead to synapse loss in neurodegenerative diseases. **(A)** Astrocytes and microglia release a number of synaptogenic factors [for example, thrombospondins (TSPs), Hevin/SPARC, Glypican 4/6, Chrdl1 from astrocytes, and brain-derived neurotrophicfactor (BDNF) and interleukin 10 (IL10) from microglia], regulating synapse formation. Astrocytes can mediate synapse pruning through MEGF10, MERTK phagocytic pathways whereas microglia contribute to synapse elimination through complement cascades [C1q, C3, and complement receptor 3 (CR3)]. Astrocytes also can regulate microglia-mediated synapse elimination by secreting transforming growth factor beta (TGFβ) and IL33. **(B)** In neurodegenerative diseases, reactive astrocytes and microglia are found around protein aggregates [such as amyloid β (Aβ) plaques, α-synuclein aggregates] and mediate synapse loss through various mechanisms. Reactive astrocytes may be induced by cytokines (IL-1α, TNF, and C1q) secreted from reactive microglia in pathological conditions, and produce neurotoxic factors that kill synapses and neurons. In addition, reactive astrocytes in Alzheimer’s disease (AD) show increased expression of glutamate transporter-1 (GLT-1), increased activity of transient receptor potential A1 (TRPA1) channels, and increased release of γ-aminobutyric acid (GABA), leading to aberrant neuronal excitability and synaptic function. Similar to astrocytes, reactive microglia can participate in neuronal damage and synapse loss through inflammatory signaling [such as TNF, IL-1β, and reactive oxygen species (ROS)]. Microglia mediate aberrant synapse loss in AD brains through complement mediators (especially C1q and C3) and triggering receptor expressedon myeloid cells 2 (TREM2).

In this review, we summarize the roles of astrocytes and microglia in synapse formation and elimination, and review/propose how mis-regulation of glial function leads to synapse loss in various neurodegenerative diseases (Figure 1).

## The Role of Glia in Synapse Formation

During CNS development, glial cells participate in regulating synapse number by inducing proper formation and elimination (Ullian et al., 2001). Over the past decades, the molecular mechanisms of glia-mediated synapse formation and elimination have been revealed.

Through *in vitro* culture of neurons, with or without astrocytes, it was initially found that astrocytes induced synapse formation by secreting several distinct molecules. When retinal ganglion cells (RGCs) were cultured in astrocyte-conditioned media (ACM), the number of synapses were increased both structurally and functionally. Later, thrombospondins (TSPs), especially TSP1 and TSP2, were found to be one of the synaptogenic proteins in the ACM. Despite effects on the formation of structural synapses, TSP1/2-induced synapses are postsynaptically silent due to their lack of functional α-amino-3-hydroxy-5-methyl-4-isoxazole propionic acid receptors (AMPARs; Christopherson et al., 2005; Eroglu et al., 2009). Along with TSPs, astrocytes express a number of matricellular proteins, such as hevin and SPARC, which modulate cell-cell and cell-matrix interactions (Eroglu, 2009). Hevin induces structurally normal and postsynaptically silent excitatory synapses, similar to TSP-induced synapses. In contrast, SPARC, a hevin homolog, antagonizes hevin and blocks synapse formation (Kucukdereli et al., 2011). Recently, it was discovered that hevin plays a role in bridging synaptic adhesion molecules neurexin 1α (NRX1α) and neuroligins (NL; Singh et al., 2016), which are localized in pre- and post-synaptic compartments, respectively (Graf et al., 2004). NLs and NRX1α, which alone are interaction-incompatible partners, can associate when transcellularly-linked by hevin. This complex can then recruit more NL1 and NMDAR to synapses (Singh et al., 2016).

So, how do astrocytes increase functional synapses? Through biochemical fractionation of ACM, glypican 4 (Gpc4) and glypican 6 (Gpc6) have been identified as functional synaptogenic molecules that strengthen glutamatergic synapses by recruiting GluA1-containing AMPARs (Allen et al., 2012). Astrocyte-secreted Gpc4 appears to upregulate release of neuronal pentraxins 1 (NP1) through interactions with presynaptic type 2a receptor protein tyrosine phosphatases δ (RPTPδ). Subsequently, NP1 binds postsynaptic AMPARs to recruit GluA1 and induce functional synapse formation (Farhy-Tselnicker et al., 2017). Astrocyte-expressed pentraxin 3 (PTX3) has been also reported to promote functionally-active CNS synapses (Fossati et al., 2019). PTX3, whose activity is regulated by TSP1, increases the surface levels and synaptic clustering of AMPARs through remodeling the perineuronal network, and a β1-integrin/ERK pathway. Chordin-like 1 (Chrdl1) has recently been shown to be another synaptogenic molecule, from astrocytes, that can induce maturation of functional synapses by increasing synaptic GluA2 AMPA receptors. Chrdl1 expression is limited to cortical astrocytes *in vivo*, and is necessary and sufficient to increase GluA2 clustering, resulting in formation of active synapses *in vitro* and *in vivo* (Blanco-Suarez et al., 2018). In addition, astrocyte-derived apolipoprotein E (APOE), which forms lipoprotein particles, with cholesterol and other lipids, has been reported to enhance presynaptic glutamatergic function (Mauch et al., 2001).

Several recent studies have suggested that microglia may also participate in inducing structural synapses. Microglia, the resident macrophages of the CNS, constantly survey and make contacts with synapses in the normal adult brain. Interestingly, when microglia were depleted by diphtheria toxin, synapse formation was disrupted, but synapse elimination rate was unchanged. Removal of brain-derived neurotrophic factor (BDNF), specifically from microglia, recapitulated this phenotype, suggesting that synapse formation is mediated by microglial BDNF (Parkhurst et al., 2013). Additionally, microglial cytokines, such as interleukin 10 (IL-10), have been shown to induce synapse formation (Lim et al., 2013). Using *in vivo* multiphoton imaging, a recent report found that microglial contact induces neuronal Ca^2+^ transients and actin accumulation, inducing filopodia formation from the dendritic branches (Miyamoto et al., 2016).

Thus, astrocytes and microglia regulate synapse formation through various mechanisms. How these different molecules engage in crosstalk, and whether neural activity/injury response controls their expression, are important questions for understanding how synapse dynamics are regulated by glial cells in healthy and diseased brains. Aberrant increases in synapse formation during development or after injury may cause hyperactive neural circuits and increased chances of epilepsy (Liuzzi and Lasek, 1987). In contrast, defective glia-mediated synapse formation could impair synaptic turnover and homeostasis, contributing to synapse loss in neurodegenerative diseases, as well as defective synaptic plasticity.

## The Role of Glia in Synapse Elimination Through Phagocytosis

To maintain proper synapse numbers, unnecessary synapses need to be eliminated during development and adulthood. Many studies have suggested that excess synapses are eliminated by neuronal activity-dependent competition (Ramiro-Cortés and Israely, 2013; Bian et al., 2015). Surprisingly, glial cells, especially astrocytes and microglia, have been shown to mediate this elimination. Astrocytes express several phagocytic receptors, such as MEGF10 (an ortholog of *Drosophila* Draper and *C. elegans* CED-1) and MERTK [a member of the Tyro-Axl-MerTK (TAM) family of receptor tyrosine kinase], and participate in eliminating synapses in the developing brain. RGCs in developing mice deficient in both *Megf10* and *Mertk* pathways show a failure of the normal refinement of connections and retain excess functional synapses with neurons in the dorsal lateral geniculate nucleus (Chung et al., 2013). This finding suggests that astrocytes actively participate in eliminating live synapses rather than simply cleaning up dead synaptic debris. Although microglia have traditionally been thought to be the major glial cells mediating synapse elimination in development, this study shows astrocytes also play a critical role. Astrocytes appear to continuously engulf both excitatory and inhibitory synapses throughout the brain during adulthood as well, suggesting that the synaptic architecture of our brains is constantly being remodeled by astrocytes in response to our experiences.

In addition to synapse elimination *via* direct phagocytosis, astrocytes also contribute to synapse elimination *via* inositol 1,4,5-triphosphate receptor type 2 (IP3R2) and *P2ry1* dependent signaling (Yang et al., 2016), as well as *via* microglia-mediated phagocytosis. Microglia mediate synapse pruning through the classical complement cascade (Schafer et al., 2012). C1q, the cascade initiating protein, is expressed by microglia and subsets of neurons (Stephan et al., 2013), and localizes to unwanted synapses for opsonization (Stevens et al., 2007). Synapse-associated C1q, then activates a downstream complement cascade and mediates microglia-dependent synapse elimination through complement receptor 3 (CR3)-mediated phagocytosis (Schafer et al., 2012). Interestingly, C1q expression in neurons is regulated by transforming growth factor (TGF)-β secreted from astrocytes (Bialas and Stevens, 2013). Additionally, it has recently been shown that microglial synapse engulfment during development is regulated by interleukin-33 (IL-33) secreted from astrocytes (Vainchtein et al., 2018), further suggesting a close interplay between astrocytes and microglia in eliminating synapses.

CX3CR1s (fractalkine receptors), expressed by microglia, also play important roles in synapse elimination. Microglia respond to neuronal fractalkine by increasing intracellular calcium transients *in vitro* and *in vivo* (Harrison et al., 1998). In *Cx3cr1*-knockout mice, migration and recruitment of microglia to the brain were impaired, resulting in an increased immature synapse population, which appeared to be responsible for weak functional synaptic connectivity and impaired social interaction in the knockout mice (Fuhrmann et al., 2010; Paolicelli et al., 2011; Hoshiko et al., 2012; Zhan et al., 2014).

## Reactive Gliosis in Response to the Injured and Diseased Brain

Reactive gliosis is observed under various conditions, such as infection, ischemia, trauma, and neurodegeneration, and usually involves hypertrophy and proliferation of glial cells and changes in gene expression. Reactive glial cells release various molecules, including chemokines, cytokines, and neurotrophic factors, that can exhibit either neuroprotective or neurotoxic effects (Sofroniew and Vinters, 2010). Although reactive gliosis was previously believed to be a secondary response to neuroinflammation, recent studies suggest that glial cells react differently depending on injury stimulus, and that gliosis can initiate neurodegeneration (Jonsson et al., 2013; Hong et al., 2016; Sekar et al., 2016).

In general, it is believed that reactive microglia can exhibit more than two polarization states, such as M1 and M2. M1-like microglia upregulate inflammatory signaling, such as TNF, IL-1β, and reactive oxygen species (ROS) signaling. In contrast, M2-like microglia express anti-inflammatory molecules, such as TGF-β1, IL-4, and IL-10. By converting their gene expression status, reactive microglia participate not only in neuronal damage and synapse loss, but also regeneration and tissue remodeling through phagocytosis (Block et al., 2007; Boche et al., 2013; Simon et al., 2017). For example, M1-like microglia led to decrease in dendritic spine density in hippocampal neurons *in vivo* and *in vitro* by producing extracellular vesicles (EVs) containing a set of miRNAs that regulate the expression of synaptic proteins. Among them, miR-146a-5p controls the expression of synaptotagmin1 and neuroligin1 in EV-receiving neurons. In contrast, M2-like microglia appear to have opposite effects by producing EVs depleted of miR-146a-5p (Prada et al., 2018).

Similar to microglia, reactive astrocytes isolated from mice with transient ischemia induced by occlusion of the middle cerebral artery (MCAO) or treated with lipopolysaccharide (LPS) differentially expressed certain genes categories depending on injury type. Genes related to metabolic activity, cell-cycle, and transcription factors (e.g., Klf5 and Klf6) were selectively increased in middle cerebral artery (MCAO)-induced reactive astrocytes (A2 astrocytes), whereas genes related to immune response, antigen processing/presentation, and complement pathway were upregulated in lipopolysaccharide (LPS)-induced reactive astrocytes (A1 astrocytes; Zamanian et al., 2012). Subsequent work has revealed that A1 astrocytes can be induced by a combination of cytokines (IL-1α, TNF, and C1q) released by reactive microglia. This study also showed that A1 astrocytes not only lose their ability to induce synapse formation and elimination, but also produce neurotoxic factors that kill neurons and oligodendrocytes (Liddelow et al., 2017). In comparison to signals inducing A1 astrocytes, little is known about the upstream inductive signals that drives A2 astrocytes. Reactive astrocytes after transient ischemic injury can transform into efficient phagocytes, clearing neuronal and synaptic debris through ATP-binding cassette transporter A1 (ABCA1)-dependent mechanisms (Morizawa et al., 2017). Furthermore, astrocytes perform neuroprotective functions through downregulating the P2Y_1_ purinergic receptor after traumatic brain injury. Downregulation of P2Y_1_ enhances extension of astrocytic processes and accelerates reactive astrogliosis accompanied by upregulation of glial fibrillary acidic protein (GFAP) and phosphorylation of STAT3. Accelerated activation of astrocytes helps diminish scar formation following injury (Shinozaki et al., 2017). Thus, these astrocytes, which presumably associated with the A2 type, may promote CNS recovery and repair (Bush et al., 1999; Gao et al., 2005; Hayakawa et al., 2014).

## Glia-Synapse Interactions in Neurodegenerative Disease

In many neurodegenerative diseases, synapse loss is a common, early sign of disease progression. As we discussed in the previous section, glia play central roles in normal synapse formation and elimination. In this section, we discuss how glial cells also participate in synapse loss in neurodegenerative diseases, especially AD and Parkinson’s disease (PD).

AD is the most common cause of dementia. Its pathology includes extracellular accumulation of Aβ plaques and intracellular, neurofibrillary tangles of hyperphosphorylated tau protein. Aβ, produced by proteolytic cleavage of amyloid precursor protein (APP), self-aggregates to form oligomers, protofibrils, or other fibrils. Soluble Aβ oligomers have been shown to induce synapse loss, tau phosphorylation, and reactive gliosis of astrocytes and microglia (Tomiyama et al., 2010; Mitew et al., 2013; Forny-Germano et al., 2014). Oligomeric Aβ surrounding senile plaques contributes to significant synapse loss in AD mouse models (Koffie et al., 2009). Although the mechanism is not fully understood, it is thought that Aβ oligomers induce overstimulation of NMDA receptors, leading to abnormal redox events and Ca^2+^ upregulation (Tu et al., 2014). Additionally, Aβ oligomers reduce synaptic adhesion by disrupting neural cell adhesion molecule 2 (NCAM2), leading to synapse loss in the AD hippocampus (Leshchyns’ka et al., 2015).

In AD brains, reactive astrocytes and microglia are found around Aβ plaques and can play beneficial or harmful roles in disease progression (Nagele et al., 2003; Olabarria et al., 2010; Simpson et al., 2010). Reactive astrocytes can internalize Aβ *in vitro* and *in vivo* (Wyss-Coray et al., 2003; Pihlaja et al., 2008), although the molecular mechanism and biological impact of this clearance are unclear. In contrast, evidence from aged AD mouse brains shows that astrocytic processes are tightly associated with Aβ plaques, but do not engulf them. Interestingly, reactive astrocytes found in AD brains seem to follow an A1 astrocyte fate, with neuroinflammatory gene expression patterns (Liddelow et al., 2017; Shi Y. et al., 2017). Since A1 astrocytes are less capable of phagocytosing neuronal material, it is possible that reactive astrocytes in AD brains become less able to engulf Aβ plaque during disease progression. Therefore, boosting the phagocytic capacity of astrocytes for Aβ plaques could lower Aβ burden in the CNS. Consistent with this hypothesis, transplantation of wild-type (WT) astrocytes into AD mouse brain results in more efficient removal of Aβ plaques compared to non-transplanted controls (Pihlaja et al., 2011). Moreover, since reactive astrocytes in AD show characteristics of A1 astrocytes, they may produce synaptic and neurotoxic factors, similar to cases of neuroinflammation and brain injury. Identifying such factors may be an important step in preventing reactive astrocyte-induced synapse loss in AD.

In AD, reactive astrocytes also directly affect synaptic function and dynamics. These astrocytes exhibit reduced expression of glutamine synthetase and glutamate transporter-1 (GLT-1; Robinson, 2001; Zumkehr et al., 2015), directly inducing aberrant neuronal excitability and synaptic function, which can lead to synapse loss. It has been suggested that reactive astrocytes can affect synaptic dysfunction through hyper-production and release of inhibitory gliotransmitter γ-aminobutyric acid (GABA). Impaired presynaptic release and spike probability, synaptic plasticity, and learning and memory were rescued by inhibiting the GABA-producing enzyme, MaoB, in AD astrocytes (Jo et al., 2014). Soluble Aβ oligomers also induce astrocytic calcium hyperactivity by activating transient receptor potential A1 (TRPA1) channels, leading to neuronal hyperactivity (Lee et al., 2014; Bosson et al., 2017). Since astrocytic calcium transients may control astrocyte-synapse interaction (Henneberger et al., 2010; Shigetomi et al., 2013; Bazargani and Attwell, 2016), dysregulation of calcium transients in AD astrocytes could be an initiating factor for synapse loss.

Similar to astrocytes, microglia are located around Aβ plaques in both human and mouse AD brains (Perlmutter et al., 1990; Bolmont et al., 2008; Grathwohl et al., 2009). It was recently found that the complement cascade that microglia use for pruning unnecessary synapses in the developing brains is responsible for synapse loss in AD brains. In Aβ overproducing AD mouse models, expression of complement mediators, especially C1q and C3, is highly upregulated, mediating aberrant synapse phagocytosis by microglia (Hong et al., 2016; Shi Q. et al., 2017). Blocking complement activation by an antibody blocking C1q function or introduction of C3^KO^ and C3R^KO^ to this AD model mouse background were sufficient to prevent early synapse loss, establishing a direct role of microglial phagocytosis in AD-associated synapse loss. A recent report also showed that in Tau-P301S transgenic mice, a C1q-blocking antibody can also prevent aberrant microglial synapse removal and rescue synaptic density (Dejanovic et al., 2018; Litvinchuk et al., 2018).

Recent studies have shown that triggering receptor expressed on myeloid cells 2 (TREM2) also plays an important role in microglia-mediated synapse elimination. Several phospholipids, including phosphatidylinositol, phosphatidylcholine, and lipoproteins (APOE, LDL, CLU/apoj), as well as Aβ, have been suggested as potential TREM2 ligands. Activation of TREM2 receptors changes microglial gene expression related to inflammatory signals and phagocytosis (Colonna, 2003). In developing brains, TREM2 maintains the phagocytic capacity of microglia and mediates normal synapse pruning (Filipello et al., 2018). Interestingly, TREM2 also mediates Aβ-induced, microglial cytokine expression and Aβ clearance through phagocytosis (Zhao et al., 2018). Determining exact functions of TREM2 in AD would be necessary in understanding the role of microglia in mediating synapse loss in AD.

PD is another common neurodegenerative disease with loss of nigrostriatal dopaminergic neurons. Abnormal intraneuronal and intraneuritic deposits of fibrillary, α-synuclein aggregates are thought to be the main cause of PD. Aggregates of α-synuclein have been found at presynaptic terminals in PD, resulting in synaptic degeneration (Schulz-Schaeffer, 2010). Previous studies demonstrated that α-synuclein induces microglial activation (Austin et al., 2006; Lee E.-J. et al., 2010) and influences microglial phagocytic activity. Park et al. (2008) showed that microglial phagocytosis was enhanced by monomeric α-synuclein, but inhibited by aggregated α-synuclein. It has been also suggested that microglia clear α-synuclein through a C1q-dependent pathway (Depboylu et al., 2011).

In PD brains, α-synuclein also stimulates reactive astrogliosis, and α-synuclein inclusions have been found in astrocytes (Wakabayashi et al., 2000; Lee H.-J. et al., 2010; Braidy et al., 2013). These studies suggest that astrocytes can uptake α-synuclein, although the physiological role of this process is unclear. Recently, it has been shown that inhibiting microglia-mediated conversion of astrocytes into A1 astrocytes significantly alleviate synaptic/neuronal loss in PD mouse models (Yun et al., 2018). Furthermore, microglia in PD show changes in expression of innate immunity genes, such as progranulin, NF-κB, and TDP-43, which may further impact microglial phagocytosis and synapse elimination (Kao et al., 2011; Frakes et al., 2014; Lui et al., 2016; Spiller et al., 2018). Therefore, similar to AD, synapse and neuronal loss in PD may be triggered by reactive microglia and astrocytes. Further studies are necessary to determine whether modulating glial function can prevent α-synuclein accumulation and synapse/neuronal loss in PD.

## Conclusions and Perspectives

We here describe recent findings that glial cells, especially astrocytes and microglia, regulate synapse dynamics in normal and neurodegenerative diseases. In the past, neurodegeneration studies have focused mainly on neurons; however, recent studies suggest that abnormal interactions between glia and synapses can initiate many pathophysiological aspects of synapse loss. In diseased brains, glial cells lose their normal functions for proper synapse formation, function, plasticity, and elimination. Furthermore, these cells acquire new, deleterious functions to drive synapse and neuronal loss. In both cases, understanding the exact molecular mechanisms responsible for glia–mediated synapse loss is an important step in developing therapeutic strategies for neurodegenerative diseases. In addition, determining what initiates reactive gliosis in each disease, and whether we can prevent these processes are equally important questions. Astrocytes and microglia may participate in synapse formation and elimination with different degrees in various development and disease conditions. In normal physiological conditions, since major portion of synapses are in direct contact with astrocytic processes, astrocytes may respond more efficiently than microglia in regulating neuronal activity-dependent changes in synaptic density. In contrast, microglia may play dominant roles in eliminating synapses in the diseased conditions due to the significant upregulation of phagocytic machineries after reactive gliosis. How astrocytes and microglia differentially and cooperatively control synapse number in the healthy and diseased brain would be additional fascinating subjects to investigate. Current advances in establishing gene expression databases of disease-affected human brains, as well as in understanding normal glial function in synapse regulation will aid this discovery.

## Author Contributions

Both authors listed have made a substantial, direct and intellectual contribution to the work, and approved it for publication.

## Conflict of Interest Statement

The authors declare that the research was conducted in the absence of any commercial or financial relationships that could be construed as a potential conflict of interest.
